# Evaluation of the *In Vitro* Antiplasmodial, Antileishmanial, and Antitrypanosomal Activity of Medicinal Plants Used in Saudi and Yemeni Traditional Medicine

**DOI:** 10.1155/2014/905639

**Published:** 2014-05-21

**Authors:** Ramzi A. Mothana, Nawal M. Al-Musayeib, Mohamed F. Al-Ajmi, Paul Cos, Louis Maes

**Affiliations:** ^1^Department of Pharmacognosy, College of Pharmacy, King Saud University, P.O. Box 2457, Riyadh 11451, Saudi Arabia; ^2^Department of Pharmacognosy, Faculty of Pharmacy, Sana'a University, P.O. Box 33039, Sana'a, Yemen; ^3^Laboratory for Microbiology, Parasitology and Hygiene (LMPH), Faculty of Pharmaceutical, Biomedical and Veterinary Sciences, Antwerp University, Universiteitsplein 1, 2610 Antwerp, Belgium

## Abstract

The antiplasmodial, antileishmanial, and antitrypanosomal activity of twenty-five medicinal plants distributed in Saudi Arabia and Yemen was evaluated. The plants were extracted with methanol and screened *in vitro* against erythrocytic schizonts of *Plasmodium falciparum*, intracellular amastigotes of *Leishmania infantum* and *Trypanosoma cruzi*, and free trypomastigotes of *T. brucei*. To assess selectivity, cytotoxicity was determined on MRC-5 cells. Criteria for activity were an IC_50_ < 10 **μ**g/mL and high selectivity (SI). Seven plants showed interesting antiprotozoal activity in one or more models. Extracts of *Caralluma penicillata* and *Acalypha ciliata* showed fairly good activity against *P. falciparum* with IC_50_ of 6.7 and 10.8 **μ**g/mL and adequate selectivity (SI > 9.6 and >5.9). Interesting activity against *L. infantum* was obtained with *Verbascum bottae* (IC_50_ of 3.2 **μ**g/mL, SI 10.2) and *Solanum glabratum* (IC_50_ 8.1 **μ**g/mL, SI 3.4). The extracts of *C. penicillata, Leucas virgata, Loranthus regularis*, and *V. bottae* exhibited moderate activity against *T. brucei* (IC_50_ 8.5, 8.1, 8.3, and 2.3 **μ**g/mL; SI > 7.6, 7.7, 4.3, and >14.1). These results partly support the traditional use of some of the selected medicinal plants and warrant further investigations into the putative active constituents.

## 1. Introduction

About one billion people are afflicted with what the World Health Organization classifies as neglected tropical diseases (NTDs). A subset of life-threatening NTDs includes Kala azar (visceral leishmaniasis), sleeping sickness (African trypanosomiasis), and Chagas disease (American trypanosomiasis) which all may lead to fatal complications but are restricted to limited geographical areas and specific groups. Most current chemotherapeutics are toxic and marginally effective, must be given by injection, and become compromised by the development of drug-resistance [1]. Despite the large numbers of people at risk and the substantial burden of disease, with few exceptions, no major interventions have been developed for generations [2]. Malaria is yet another even bigger threat in many parts of the world with resistance spreading to almost all classes of antimalarials [3]. Hence, safe, effective, and more affordable therapeutics are clearly needed, whereby the natural biodiversity with its numerous plants, microorganisms, and marine organisms constitutes a broad source of potentially new molecules with great variety of structures and pharmacological potential. In addition, it is estimated that two-thirds of the world population still rely on traditional medical remedies, mostly plants, because of limited availability or affordability of pharmaceutical medicines [4].

In the present study, we evaluated twenty-five medicinal plants used in traditional medicine in Yemen and Saudi Arabia for their* in vitro* antiplasmodial, antileishmanial, and antitrypanosomal potential.

## 2. Materials and Methods

### 2.1. Plant Material

Twenty-five plants were selected randomly from different areas of Yemen and Saudi Arabia in March and June 2008 and were identified at the Pharmacognosy Departments, Colleges of Pharmacy, King Saud and Sana'a Universities, Saudi Arabia and Yemen. The plants were chosen mainly on the basis of their local medicinal knowledge. Voucher specimens were deposited at the departments. The botanical names, plant parts used, and the traditional uses in the collected areas are presented in Table 1.

### 2.2. Extraction of Plant Materials

The air-dried and powdered plant material (50 g) was extracted with 500 mL methanol (CH_3_OH) using a Soxhlet apparatus for 8 hours. The obtained methanolic extract was filtered and evaporated in a rotator evaporator and freeze dryer. The dried extracts were stored at −20°C until used. Stock solutions were prepared in 100% DMSO at 20 mg/mL just prior to* in vitro* evaluation.

### 2.3. Reference Drugs

For the different tests, appropriate reference drugs were used as positive control: tamoxifen for MRC-5, chloroquine for* Plasmodium falciparum*, miltefosine for* Leishmania infantum*, benznidazole for* Trypanosoma cruzi,* and suramin for* Trypanosoma brucei*. All reference drugs were obtained either from the fine chemical supplier Sigma or from WHO-TDR.

### 2.4. Biological Assays

The integrated panel of microbial screens and standard screening methodologies were adopted as previously described [8]. All assays were performed at the Laboratory of Microbiology, Parasitology and Hygiene at the University of Antwerp, Belgium. Plant extracts were tested in triplicate at 5 concentrations (64, 16, 4, 1, and 0.25 *μ*g/mL) to establish a full dose-titration and determination of the IC_50_ (inhibitory concentration 50%). The in-test concentration of DMSO did not exceed 0.5%.

#### 2.4.1. Antileishmanial Activity

*L. infantum* MHOM/MA (BE)/67 amastigotes were collected from the spleen of an infected donor hamster and used to infect primary peritoneal mouse macrophages. Macrophages (3 × 10^4^) were seeded in each well of a 96-well plate. After 2 days of outgrowth, 5 × 10^5^ amastigotes were added to each well and incubated for 2 h at 37°C. The prediluted extracts were subsequently added and the plates were further incubated for 5 days at 37°C and 5% CO_2_. Intracellular amastigotes burdens were microscopically assessed after Giemsa staining and expressed as a percentage of the burdens in the blank controls.

#### 2.4.2. Antiplasmodial Activity

Chloroquine-resistant* P. falciparum* K1-strain was grown in human erythrocytes O^+^ at 37°C under a low oxygen atmosphere (3% O_2_, 4% CO_2,_ and 93% N_2_) in RPMI-1640 medium supplemented with 10% human serum. Infected human red blood cells (200 *μ*L, 1% parasitaemia and 2% hematocrit) were added to each well containing the prediluted extracts and incubated for 72 h. After incubation, test plates were frozen at −20°C and parasite multiplication was measured by the Malstat method [9].

#### 2.4.3. Antitrypanosomal Activity

*T. brucei* Squib-427 strain (suramin-sensitive) was cultured at 37°C and 5% CO_2_ in Hirumi-9 medium [10] supplemented with 10% fetal calf serum (FCS). About 1.5 × 10^4^ trypomastigotes were added to each well and parasite growth was assessed after 72 h at 37°C by adding resazurin [11]. For Chagas disease,* T. cruzi* Tulahuen CL2 (benznidazole-sensitive) was maintained on MRC-5 cells in minimal essential medium (MEM) supplemented with 20 mM L-glutamine, 16.5 mM sodium hydrogen carbonate, and 5% FCS. In the assay, 4 × 10^3^ MRC-5 cells and 4 × 10^4^ parasites were added to each well. After incubation at 37°C for 7 days, parasite growth was assessed by adding the *β*-galactosidase substrate chlorophenol red *β*-D-galactopyranoside [12]. The color reaction was read at 540 nm after 4 h and absorbance values were expressed as a percentage of the blank controls.

#### 2.4.4. Cytotoxicity Assay

MRC-5_SV2_ cells were cultivated in MEM supplemented with L-glutamine (20 mM), 16.5 mM sodium hydrogen carbonate, and 5% FCS. For the assay, 10^4^ MRC-5 cells/well were seeded onto the test plates containing the prediluted extracts and incubated at 37°C and 5% CO_2_ for 72 h. Cell viability was assessed fluorimetrically after 4 hours of addition of resazurin. Fluorescence was measured (excitation 550 nm, emission 590 nm) and the results are expressed as % reduction in cell viability compared to the blank controls.

## 3. Results

For the selection of relevant plant species for our screening program, different places in Yemen and Saudi Arabia were visited where elderly people with profound knowledge of folk medicine were interviewed. In total, 25 medicinal plants were collected (Table 1), extracted, and evaluated in the integrated* in vitro* screen for antileishmanial, antiplasmodial, and antitrypanosomal potential (Table 2). Seven plants showed interesting activity (acceptable potency and selectivity) in one or more models (Table 2).

### 3.1. Antiplasmodial Activity

The methanolic extracts of* Caralluma penicillata* and* Acalypha ciliata* showed relevant activity against* P. falciparum* with IC_50_ of 6.7 and 10.8 *μ*g/mL, respectively, and acceptable selectivity (SI > 9.6 and > 5.9). The extracts of* Dichrocephala integrifolia* and* Rosmarinus officinalis* showed similar activity with IC_50_ values of 10.2 and 11.4 but with low selectivity (SI = 2.4 and 1.9).* Hypoestes forskalei* demonstrated nonselective inhibition (IC_50_ 8.8 *μ*g/mL; SI 1.3).

### 3.2. Antileishmanial Activity

The extract of* Verbascum bottae* showed promising and selective activity against* L. infantum* (IC_50_ 3.2 *μ*g/mL; SI 10.2);* Solanum glabratum* exhibited moderate antileishmanial activity (IC_50_ 8.1 *μ*g/mL; SI 3.4).* H. forskalei* demonstrated nonselective inhibition (IC_50_ 8.1 *μ*g/mL; SI 1.4).

### 3.3. Antitrypanosomal Activity

Against* T. brucei*, the extracts of* C. penicillata*,* Leucas virgata*,* Loranthus regularis,* and* V. bottae* exhibited the most remarkable activity with IC_50_ values of 8.5, 8.1, 8.3, and 2.3 *μ*g/mL, respectively, and acceptable selectivity (SI > 7.6, 7.7, 4.3, and >14.1). Against* T. cruzi*, some inhibitory potential was shown for* D. integrifolia*,* R. officinalis,* and* S. glabratum*, however, with lower selectivity (IC_50_ 6.6, 8.8, and 8.5 *μ*g/mL, resp., with SI < 4).

### 3.4. Cytotoxicity

The selectivity of the antiprotozoal activity was assessed on MRC-5 cells. Clear cytotoxicity was found for* Gomphocarpus fruticosus* (IC_50_ 2.3 *μ*g/mL),* Centaurothamus maximus* (IC_50_ 9.3 *μ*g/mL), and* Hypoestes forskalei *(IC_50_ 11.0 *μ*g/mL). The observed antiprotozoal inhibition of these plant species is therefore considered as nonselective.

## 4. Discussion

In continuation of our search for substances of plant origin with pharmacological effects, 25 plants were collected from Saudi Arabia and Yemen and screened for their antiplasmodial, antileishmanial, and antitrypanosomal activity potential. It is important to mention that to the best of our knowledge, this study represents the first report on antiprotozoal evaluation for most of the investigated plants. Although some plants have already been partly investigated, knowledge remains very limited in many cases. Based on potency (IC_50_) and selectivity, seven plant extracts are considered promising enough to pursue further purification and biological evaluation of individual constituents.

The methanol extract of the* C. penicillata* (collected from Saudi Arabia) exhibited antiplasmodial activity with adequate selectivity (IC_50_ 6.7 *μ*g/mL, SI 9.6). Some side-activity was present against* T. brucei* (IC_50_ 8.5 *μ*g/mL, SI 7.6), which matches our previously published data on* C. sinaica* showing antileishmanial and antitrypanosomal activity. However,* C. sinaica* was inactive against* P. falciparum* [13]. Pregnane glycosides which represent the major compounds in* Caralluma* species are believed to be responsible for the observed effects. Isolation and characterization of some acylated pregnane glycosides revealed antiparasitic activity for* C. tuberculata* and* C. penicillata*. The pregnane glycosides penicilloside E isolated from* C. penicillata* and caratuberside C isolated from* C. tuberculata* exhibited a pronounced antitrypanosomal activity (IC_50_ 1.0 and 1.8 *μ*g/mL) [14, 15].

Interesting antiplasmodial activity was obtained with* A. ciliata* that is traditionally used in the treatment of malaria. Our result is in agreement with literature data on other* Acalypha* species [16–20]. We previously reported interesting antiplasmodial activity for the methanol and aqueous extracts of* A. fruticosa* [16], whereby both extracts showed complete inhibition of schizont maturation at 7.8 *μ*g/mL. Furthermore, Bradacs et al. [17] reported that the leaf extracts of* A. grandis* significantly affected* P. falciparum* without showing obvious effects on other protozoa. Additionally, the extract and fractions of* A. wilkesiana* dose-dependently reduced parasitaemia induced by chloroquine-sensitive* P. berghei* infection in prophylactic, suppressive, and curative mouse models [18].

Interesting antileishmanial and antitrypanosomal activities were observed for* S. glabratum*. Antiprotozoal properties have indeed been reported for extracts from other* Solanum* species [21–25]. Abdel-Sattar et al. [21] demonstrated potent* in vitro* antitrypanosomal activity for the methanol extract of* S. schimperianum* (IC_50_ 0.061 *μ*g/mL). It is recently reported that the extract of* S. torvum* inhibited the proliferation of promastigotes of* L. donovani* [22]. The fruits of* S. stramonifolium* var.* stramonifolium* were shown to have marginal activity against amastigotes of* L. amazonensis* [23]. Although* S. sisymbriifolium* failed to inhibit promastigotes of* L. amazonensis* and* L. brasiliensis* (IC_50_ of 33.8 and 20.5 *μ*g/mL), the steroid derivative Cilistol-A as the main active principle of the chloroform fraction exhibited significant activity against both* Leishmania* species (IC_50_ 6.6 and 3.1 *μ*g/mL) [24]. It seems that the antileishmanial as well as antitrypanosomal activities can be attributed to the steroidal alkaloids, which represent the major constituents in* Solanum* species. Abreu Miranda et al. [25] isolated solamargine and solasonine from the fruits of* S. lycocarpum* and showed* in vitro* leishmanicidal activity against promastigotes of* L. amazonensis*. Our failure to demonstrate activity against* P. falciparum* (IC_50_ > 64 *μ*g/mL) is not in agreement with literature data on other* Solanum* species. For example, Chinchilla et al. [26] reported antimalarial effect for* S. arboretum*; Kamaraj et al. [27] found some effect for* S. torvum* against chloroquine-sensitive (3D7) and chloroquine-resistant strains of* P. falciparum*. Moreover, diosgenone which is a spirostan-type steroidal saponin isolated from* S. nudum*, showed a high activity against FCB-2 strain of* P. falciparum* [28]. These results showed that diosgenone could be a new therapeutic alternative for the treatment of malaria [28].

One of the more remarkable plants with antileishmanial and antitrypanosomal activities was* V. bottae*, which showed selective activity against* L. infantum* and* T. brucei* (IC_50_ 3.2 and 2.3 *μ*g/mL; SI 10.2 and 14.1). This result outperforms data reported on other* Verbascum* species. A very marginal antileishmanial activity for* V. arcturus* against* L. donovani* (IC_50_ 57 *μ*g/mL) was reported and no activity against* P. falciparum* was reported [29]. Manjili et al. [30] obtained an IC_50_ of 451 *μ*g/mL for a* V. thapsus* extract against* L. major* promastigotes, illustrating inefficacy.

Another plant with antitrypanosomal activity was* L. virgata* (IC_50_ 8.8 *μ*g/mL against* T. brucei*), which is in agreement with literature data on other* Leucas* species [31–34]. Our results on the antiplasmodial inactivity of* L. virgata* are not in agreement with the effects noted for* L. aspera* and* L. cephalotes*. It is recently reported that the leaf ethyl acetate and methanol extracts of* L. aspera* had good antiplasmodial activity (IC_50_  7.81 and 22.7 *μ*g/mL with SI values of 5.4 and 2.0) [32]. A similar study was conducted by Kamaraj et al. [33] who reported similar results for* L. aspera* (IC_50_ 12.5 *μ*g/mL). In addition, it is reported that* L. cephalotes* showed promising antiplasmodial activity (IC_50_ < 5 *μ*g/mL) in addition to promising activities against* L. donovani* with IC_50_ values of 3.61 *μ*g/mL (SI = 8) [34]. Apparently, these findings are mostly attributed to the presence of essential oil constituents as well as diterpenoids [35–38]. Triterpenoids, such as ursolic acid isolated from some* Leucas* species [39], showed significant antitrypanosomal activity: it inhibited all movement of* T. cruzi* epimastigotes at 40 *μ*g/mL after 48 h incubation [40].

Notably antitrypanosomal potencies against* T. brucei* were also displayed by the methanolic extract of* L. regularis* (IC_50_ 9.5 *μ*g/mL, SI 4.3). Based on the literature review and to the best of our knowledge this is the first report on antiprotozoal activity of the genus* Loranthus*.

The methanolic extract of* R. officinalis* inhibited both* P. falciparum* and* T. cruzi* (IC_50_ 11.4 and 8.8 *μ*g/mL; SI 1.9 and 2.5) which is in agreement with data reported previously [35, 40]. The inhibitory effect was attributed to the presence of essential oils and triterpenoids, such as ursolic acid and oleanolic acid [40].

## 5. Conclusion

In conclusion, this preliminary study led to the identification of seven plant extracts, namely,* A. ciliata*,* C. pencillata*,* L. virgata*,* L. regularis*,* R. officinalis*,* S. glabratum,* and* V. bottae* exhibiting relevant antiplasmodial, antileishmanial, and antitrypanosomal activity in one or more models. The obtained results support to some extent the traditional uses of some plants for the treatment of parasitic diseases. Isolation, purification, and structure elucidation of constituents from some of these investigated plants are warranted to support discovery of novel antiplasmodial, antileishmanial, and antitrypanosomal compounds.

## Figures and Tables

**Table 1:** List of selected plants and their use in traditional medicine.

Plant species	Family	Part used	Traditional uses
*Acalypha ciliata *Forssk.	Euphorbiaceae	L	Malaria, anthelmintic, and scabies (a, d)
*Acalypha fruticosa *Forssk.	Euphorbiaceae	L	Skin diseases, malaria, and wounds (a, b, d)
*Amaranthus viridis* L.	Amaranthaceae	L	Anthelmintic, cancer, and impotence (a, b)
*Barleria trispinosa *(Forssk.) Vahl	Acanthaceae	L, S	Warts (a, d)
*Caralluma penicillata *(Deflers) N.E.Br.	Asclepiadaceae	L	Diabetes, stomach ulcer, and smallpox (a, c)
*Centaurothamus maximus *(Forssk.)	Asteraceae	L	Wounds (a)
Wagenitz & Dittrich			
*Cissus rotundifolia *(Forssk.) Vahl	Vitaceae	L	Malaria, liver disease, and otitis (a, b, c, d)
*Coccinia grandis *(L.) Voigt	Cucurbitaceae	L, T	Anthelmintic, diuretic, and pneumonia (a)
*Dichrocephala integrifolia *(L.f.) O.	Asteraceae	L, S	Wounds (a)
Kuntze			
*Fagonia indica* Burm. f.	Zygophyllaceae	L, T	Diabetes, diuretic, and headache (a, b, c)
*Forsskaolea tenacissima *L.	Urticaceae	L, S	Diuretic, kidney disease (a)
*Gomphocarpus fruticosus (L.) Ait. f. *	Asclepiadaceae	L	Tumors, skin disease, scabies, and itching (a, b)
*Hypoestes forskalei (Vahl) R. Br. *	Acanthaceae	L	Fungal skin disease, scabies, itching, and warts (a)
*Kedrostis foetidissima*(Jacq.) Cogn.	Cucurbitaceae	L, S	Warts (a)
*Kleinia pendula *(Forssk.) DC.	Asteraceae	R	Otitis (a)
*Leucas virgata *	Labiatae	L, T	Heartburn, indigestion, and stomach problems (a)
*Loranthus regularis* Steud. ex Sprague	Loranthaceae	R	Diabetes, kidney disease (a)
*Ochradenus baccatus *Del.	Resedaceae	L, F	Diuretic, antiseptic, cough, and itching (a, b)
*Otostegia fruticosa* (Forssk.) Briq.	Labiatae	L, F	Antiparalytic, eye diseases (a)
*Oxalis corniculata* L.	Oxalidaceae	L, F	Antiparasitic, antivertigo, and mouth inflammation (a, b, c)
*Rosmarinus officinalis* L.	Labiatae	L, S	Antiseptic, cholagogue (a, d)
*Solanum glabratum* Dunal	Solanaceae	L, T	Diuretic, scabies, syphilis, cough, hemorrhoids (a, b, c, d)
*Taraxacum officinale* F.H. Wigg	Asteraceae	L	Gastrointestinal troubles (a)
*Tecoma stans* (L.) H.B.K.	Bignoniaceae	L, S	Diabetes (a, b)
*Verbascum bottae* (Deflers) Huber-Mor.	Scrophulariaceae	L, F	Cough, skin disease, and rheumatism (a, b)

F: flower, L: leaves, R: roots or rhizomes, S: stems, and T: fruits. a: information has been taken from native people; b: Al-Dubai and Al-Khulaidi (1996) [5]; c: Fleurentin and Pelt (1982) [6]; d: Schopen (1983) [7].

**Table 2:** Antiprotozoal activity and cytotoxicity (IC_50_—*μ*g/mL) of the methanolic extracts of the investigated plants.

Plant species	*P. falciparum *		*L. infantum *		*T. cruzi *		*T. brucei *		MRC-5
	IC_50_	SI	IC_50_	SI	IC_50_	SI	IC_50_	SI	IC_50_
*Acalypha ciliata *	**10.8 ± 1.3 **	>5.9	>64.0		34.4 ± 7.3	>1.9	32.7 ± 3.7	>2.0	>64.0
*Acalypha fruticosa *	27.1 ± 6.2	>2.4	>64.0		35.7 ± 5.8	>1.8	32.9 ± 4.1	>2.0	>64.0
*Amaranthus polygamus *	>64.0	>1	>64.0		>64.0		>64.0		>64.0
*Barleria trispinosa *	59.8 ± 5.6	>1.1	>64.0		>64.0		>64.0		>64.0
*Caralluma penicillata *	**6.7 ± 0.9**	>9.6	34.6 ± 4.5	>1.9	28.0 ± 1.7	>2.3	**8.5 ± 1.5**	>7.6	>64.0
*Centaurothamus maximus *	12.1 ± 3.4	<1	32.5 ± 0.5	<1	8.3 ± 0.7	1.1	35.8 ± 2.3		**9.3 ± 1.8**
*Cissus rotundifolia *	>64.0	>	>64.0		>64.0		>64.0		>64.0
*Coccinia grandis-fruits *	>64.0	>	>64.0		>64.0		>64.0		>64.0
*Coccinia grandis-leaves *	38.6 ± 7.3	>1.7	43.1 ± 4.8	>1.5	36.9 ± 2.4	>1.7	32.7 ± 4.3	>2	>64.0
*Dichrocephala integrifolia *	10.2 ± 2.5	2.4	20.3 ± 2.6	1.2	**6.6 ± 0.5**	3.7	37.6 ± 4.9		24.5 ± 3.7
*Fagonia indica *	>64.0		>64.0		>64.0		>64.0		>64.0
*Forsskaoleatenacissima *	>64.0		>64.0		>64.0		>64.0		>64.0
*Gomphocarpus fruticosus *	>64.0		>64.0		2.6 ± 0.5	1	>64.0		**2.3 ± 0.4**
*Hypoestes forskalei *	8.8 ± 2.0	1.3	8.1 ± 1.3	1.4	9.1 ± 1.0	>1.2	8.1 ± 0.5	>1.4	**11.0 ± 2.1**
*Kedrostis foetidissima *	62.2 ± 8.3	>1	>64.0		60.2 ± 3.5	>1.1	>64.0		>64.0
*Kleinia pendula *	38.9 ± 6.4	>1.6	>64.0		32.3 ± 3.7	>2	32.7 ± 4.0	>2	>64.0
*Leucas virgata *	>64.0		>64.0		>64.0		**8.3 ± 0.9**	>7.7	>64.0
*Loranthus regularis *	>64.0		32.5 ± 0.5	1.3	33.6 ± 2.5	1.2	**9.5 ± 2.1**	4.3	40.6 ± 5.5
*Ochradenus baccatus *	>64.0		>64.0		>64.0		>64.0		>64.0
*Otostegia fruticosa *	34 ± 5.2	>1.9	>64.0		36.6 ± 4.9	>1.8	35.2 ± 3.7	>1.8	>64.0
*Oxalis corniculata *	>64.0		>64.0		54.7 ± 7.6	>1.2	34.7 ± 5.2	>1.8	>64.0
*Rosmarinus officinalis *	**11.4 ± 2.7**	1.9	32.5 ± 0.5		**8.8 ± 1.2**	2.5	13.2 ± 2.0	1.7	22.1 ± 1.7
*Solanum glabratum *	>64.0		**8.1 ± 1.3**	3.4	**8.5 ± 0.9**	3.3	30.0 ± 4.8		27.9 ± 3.4
*Taraxacum officinale *	>64.0		>64.0		45.6 ± 7.3	>1.4	>64.0		>64.0
*Tecoma stans *	36.3 ± 5.8	>1.8	38.1 ± 4.7	>1.7	31.7 ± 3.7	>2.0	36.9 ± 6.3	>1.7	>64.0
*Verbascum bottae *	29.9 ± 4.5	1.1	3.2 ± 0.3	10.2	27.0 ± 4.2	1.2	**2.3 ± 0.9**	14.1	32.5 ± 5.9
Chloroquine	0.3 ± 0.1								
Miltefosine			3.32 ± 0.7						
Benznidazole					2.2 ± 0.5				
Suramin							0.03 ± 0.02		
Tamoxifen					—				11.0 ± 2.3

IC_50_ values of reference drugs are expressed in *μ*M concentrations.
